# Three Novel Triterpenoids from *Taraxacum officinale* Roots

**DOI:** 10.3390/molecules21091121

**Published:** 2016-08-27

**Authors:** Takashi Kikuchi, Ayaka Tanaka, Mayu Uriuda, Takeshi Yamada, Reiko Tanaka

**Affiliations:** Laboratory of Medicinal Chemistry, Osaka University of Pharmaceutical Sciences, 4-20-1 Nasahara, Takatsuki, Osaka 569-1094, Japan; t.kikuchi@gly.oups.ac.jp (T.K.); e11531@gap.oups.ac.jp (A.T.); cocoon.020217@gmail.com (M.U.); yamada@gly.oups.ac.jp (T.Y.)

**Keywords:** 18β,19β-epoxy-21β-methoxylupan-3β-yl acetate, 3β-acetoxybauer-7-en-6-one, 3β-acetoxyeuph-7,24-dien-6-one, *Taraxacum officinale*, dandelion, triterpene, NO production

## Abstract

Three novel lupane-, bauerane-, and euphane-type triterpenoids (**1**–**3**), in addition to seven known triterpenoids (**4**–**10**)—18β,19β-epoxy-21β-hydroxylupan-3β-yl acetate (**4**), 21-oxolup-18-en-3β-yl acetate (**5**), betulin (**6**), officinatrione (**7**), 11α-methoxyolean-12-en-3-one (**8**), eupha-7,24-dien-3-one (**9**), and 24-oxoeupha-7,24-dien-3β-yl acetate (**10**)—were isolated from the roots of *Taraxacum officinale*. Their structures were elucidated on the basis of spectroscopic analyses using 1D and 2D-NMR spectra and electron ionization mass spectrometry (EIMS). The effects of compounds **1**–**10** on the production of nitric oxide (NO) in lipopolysaccharide (LPS)-activated mouse peritoneal macrophages were evaluated. Compounds **4**, **6**, and **10** exhibited similar NO inhibitory activities to N^G^-monomethyl-l-arginine acetate (l-NMMA). These compounds did not exhibit cytotoxicity at an effective concentration. The results of present study suggest that compounds **4**, **6**, and **10** have potential as anti-inflammatory disease agents.

## 1. Introduction

**Figure 1:**
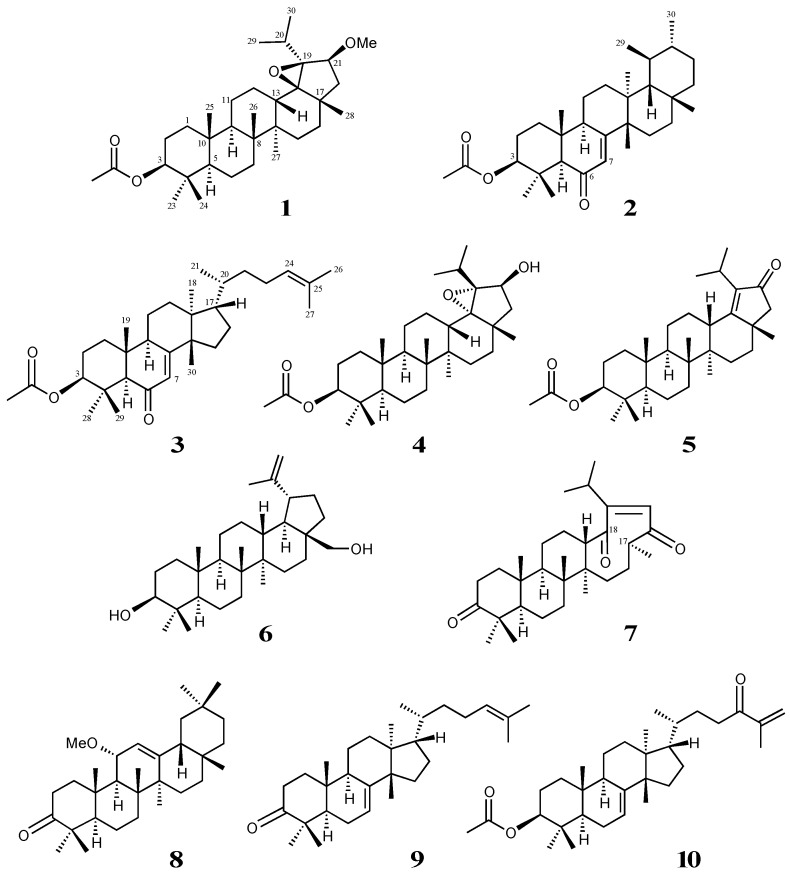
Chemical structures for compounds **1**–**10**.

*Taraxacum officinale* (commonly known as dandelion) has a number of culinary and medicinal uses, despite being generally regarded as a weed. Dandelion is the general name of approximately two thousand species of plants that belong to the genus *Taraxacum* (Compositae). Young leaves and inflorescences are used as ingredients in salads and stir-fries. Dandelion roots have long been largely used on the continent, and the plant is cultivated largely in India as a remedy for liver complaints. Dandelion roots contain bitter principles that have a tonic effect on the liver and digestive system [1]. It is also a gentle laxative and natural diuretic that is rich in natural potassium, which enriches the body′s supply. It has been used as a tonic to treat rheumatic issues and also acts as a blood purifier [2]. In recent studies, it was revealed that dandelion extract may play a significant role during adipogenesis and lipid metabolism, and may have the potential for the treatment of obesity [3]. Dandelion is available as capsules, liquid extracts, and tea forms [4]. We previously reported five novel lupan-type triterpenoids isolated from the roots of *T. officinale.* Of these, officinatrione, a (17*S*)-17,18-*seco*-lupane was identified as a new carbon skeletal triterpenoid [5]. We herein reported three new triterpenoids: 18β,19β-epoxy-21β-methoxylupan-3β-yl acetate (**1**), 3β-acetoxybauer-7-en-6-one (**2**), 3β-acetoxyeupha-7,24-dien-6-one (**3**), in addition to seven known compounds (**4**–**10**) (Figure 1). The isolated compounds (**1**–**10**) were evaluated for their inhibitory activities on macrophage activation using an inhibitory assay of nitric oxide (NO) production in RAW 264.7 mouse macrophages stimulated by lipopolysaccharide (LPS).

## 2. Results and Discussion

The methanol extract of the roots of *T. officinale* (5.81 kg) was partitioned between EtOAc and water. The organic layer was condensed in vacuo to give a yellowish residue (890 g) and separated by silica gel chromatography followed by HPLC [octadecyl silica (ODS), MeOH–H_2_O (95:5)] to afford the novel lupane, bauerane, euphane-type triterpenes, as well as seven known triterpenes.

**Figure 2:**
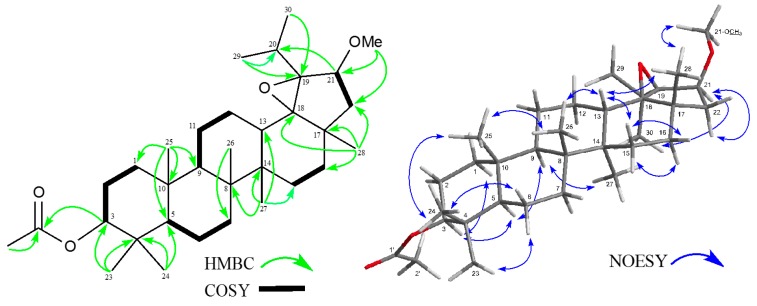
Key HMBC, COSY and NOESY correlations for **1**.

Compound **1** was obtained as colorless needles, the molecular ion peak at *m/z* 514.4022 in high resolution (HR) electron ionization (EIMS) of which showed the molecular formula C_33_H_54_O_4_ (calcd. for 514.4022). Its IR spectrum showed the presence of an ester group at 1739 and 1246 cm^−1^. Detailed assignments of ^1^H- and ^13^C-NMR spectra (Table 1) using hetero-nuclear single quantum coherence (HSQC) and distorsionless enhancement by polarization transfer (DEPT) methods revealed the presence of six tertiary methyls (δ_H_ 0.84, 0.85, 0.88, 1.05, 1.075 and 1.083 (each 3H, s)), two secondary methyls (δ_H_ 1.11 (6H, d, *J* = 7.4 Hz)), an acetyl methyl (δ_H_ 2.04 (s), δ_C_ 171.0 (s)), nine methylenes, six methines including two oxymethines (δ_H_ 3.77 (d, *J* = 5.9 Hz), δ_C_ 83.2 (d); δ_H_ 4.47 (dd, *J* = 11.4, 5.3 Hz), δ_C_ 80.9 (d)), five quarternary carbons, and two oxygenated sp^3^ quaternary carbons (δ_C_ 71.9 (s), 77.0 (s)). EIMS showed fragment ion peaks characteristic of the cleavage of the C-ring at *m/z* 249 (C_16_H_25_O_2_) and 189 (249 − HOAc), which revealed the presence of an acetyl group at the C-3 position [6]. The planar structure of **1** was established using the hetero-nuclear multiple quantum coherence (HMBC) spectrum, and selected HMBC correlations are shown in Figure 2. Strong correlations were observed between Me-23, 24 (δ_H_ 0.85, 0.84, respectively) and C-3 (δ_C_ 80.9), C-4, and C-5; between Me-25 (δ_H_ 0.88) and C-1, C-5, C-9, and C-10; between Me-26 (δ_H_ 1.075) and C-7, C-8, C-9, and C-14; between Me-27 (δ_H_ 1.05) and C-8, C-13, C-14, and C-15; between Me-28 (δ_H_ 1.083) and C-16, C-17, C-18 (δ_C_ 77.0), and C-22; between Me-29, 30 (δ_H_ 1.11 (6H)) and C-19 (δ_C_ 71.9), and C-20 (δ_C_ 29.4); between H-13 (δ_H_ 2.48) and C-11, C-12, C-14, C-15, C-17, C-18, C-19, and C-27; between H-21 (δ_H_ 3.77) and C-17, C-18, C-19, C-20, and C-22; and between OMe (δ_H_ 3.22) and C-21. In the ^1^H-^1^H correlation spectroscopy (COSY) spectrum, cross peaks were observed between H-21 (δ_H_ 3.77) and H_2_-22 (δ_H_ 1.18, 1.32) and also between H-3 and H_2_-2 (δ_H_ 1.64 (2H)), and other cross peaks are shown by bold-faced lines in Figure 2. The relative configuration was established by the nuclear overhauser effect correlated spectroscopy (NOESY) spectrum (Figure 2); between H-3α (δ_H_ 4.47) and H-5α (δ_H_ 0.81) and Me-23 (δ_H_ 0.85); between H-5α (δ_H_ 0.81) and H-9α (δ_H_ 1.38); between Me-24 (δ_H_ 0.84) and Me-25 (δ_H_ 0.88); between Me-26 (δ_H_ 1.075) and Me-28 (δ_H_ 1.083) and H-13β (δ_H_ 2.48); between Me-27 (δ_H_ 1.05) and H-9α; between Me-28 (δ_H_ 1.083) and Me-26 and OMe (δ_H_ 3.22); between Me-29 (δ_H_ 1.11) and H-21α (δ_H_ 3.77) and OMe; and between H-21 (δ_H_ 3.77) and Me-29 and H-22α. However, a cross peak was not observed between Me-28 and H-21. Therefore, the 18,19-epoxy ring and secondary methoxy group at the C-21 in the β orientation and the structure of **1** was elucidated as 18β,19β-epoxy-21β-methoxylupan-3β-yl acetate.

**Figure 3:**
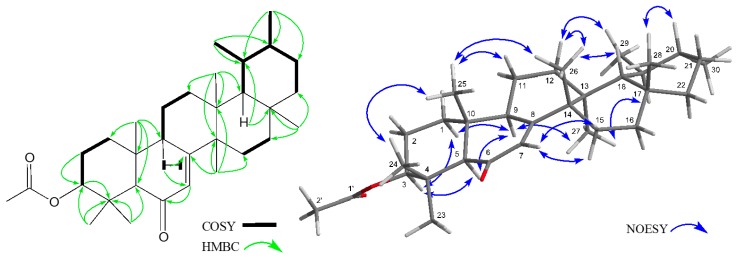
Key HMBC, COSY and NOESY correlations for **2**.

Compound (**2**) was obtained as colorless crystals, and its molecular formula was established as C_32_H_50_O_3_ ([M]^+^; *m*/*z* 482.3759, calcd. for 482.3760) by HREIMS. IR and UV spectra revealed the presence of an ester group and an αβ-unsaturated six-membered ring ketone at ν_max_ 1733, 1247, and 1665 cm^−1^ and λ_max_ at 245.0 nm (log ε 3.97). The ^1^H- and ^13^C-NMR spectra of **2** (Table 2) indicated the presence of six tertiary methyls (δ_H_ 0.88, 0.96, 1.06 (6H), 1.19, 1.20 (each s)), two secondary methyls (δ_H_ 0.92 (d), 1.07 (d)), an acetyl methyl (δ_H_ 2.07 (s)), eight methylenes, six methines including an oxymethine (δ_H_ 4.47 (dd), δ_C_ 80.6 (d)), five sp^3^ quaternary carbons, and an αβ-unsaturated six-membered ring ketone (δ_H_ 5.84 (d), δ_C_ 123.9 (d), 170.3 (s), 199.8 (s)). In the ^1^H-^1^H COSY spectrum, correlations of H_2_-1–H-3; H-9–H_2_-12; H_2_-15–H_2_-16; and H-18–H_2_-22 were observed (Figure 3). The planar structure of **2** was established by a comprehensive analysis of 1D and 2D NMR spectra, particularly the HMBC spectrum. Selected HMBC correlations are shown in Figure 3. Strong correlations were observed from Me-23 (δ_H_ 1.20 (s)) and Me-24 (δ_H_ 1.19 (s)) to C-3 (δ_C_ 80.6 (d)), C-4 and C-5; from Me-25 (δ_H_ 0.88 (s)) to C-1, C-5, C-9 and C-10; from Me-26 (δ_H_ 1.06 (s)) to C-8 (δ_C_ 170.3 (s)), C-13, C-14 and C-15; from Me-27 (δ_H_ 0.96 (s)) to C-12, C-13, C-14 and C-17; from Me-28 (δ_H_ 1.06 (s)) to C-16, C-17, C-18 and C-22; from Me-29 (δ_H_ 1.07 (d)) to C-18, C-19 and C-20; from Me-30 (δ_H_ 0.92 (d)) to C-19, C-20 and C-21; from H-7 (δ_H_ 5.84 (d)) to C-5, C-6 (δ_C_ 199.8 (s)), C-8, C-9 and C-14; and from H-18 (δ_H_ 1.33) to C-12, C-13, C-14, C-18, C-19, C-20, C-27 and C-28. Based on the above spectral data, in addition to the ^1^H-NMR chemical shift in **2** compared with previous data on baurerenyl acetate [7,8], the plain structure was assumed to be *D:C*-friedours-7-ene-6-one (baueran-7-en-6-one). The relative configuration of **2** was established in the NOESY experiment. Strong NOESY correlations between Me-23 and H-3α; between Me-25 and Me-24 and Me-26; between Me-27 and H-9α and Me-30; between H-5α and H-9α: between H-7 and H-15α and H-15β; between H-18 and Me-26, Me-28 and Me-29; between H-19 and Me-27; and between H-20 and Me-28 revealed an acetyl group at C-3 in the β orientation, H-18 in the β orientation, and Me-19 and Me-20 in the β and α orientations, respectively. Therefore, the structure of **2** was established as 3β-acetoxybauer-7-en-6-one. Natori et al. synthesized 3β-acetoxy-9α-bauer-7-en-6-one (XIII) from the oxidation of bauerenyl acetate, and confirmed the structure of XIII from a comparison between 3β-acetoxy-9β-bauer-7-en-6-one (XII) and XIII [9]. However, a marked difference was found in the ^1^H-NMR chemical shifts in **2** and XIII; therefore, the structure of XIII does not appear to be reasonable due to the lack of sufficient evidence. On the other hand, Yen isolated 3β-acetoxy-9β-bauer-7-en-6-one from the stem bark of *Hiptage benghalensis*, and the chemical shift value of ^1^H-NMR was similar to that described above for compound XII [10].

**Figure 4:**
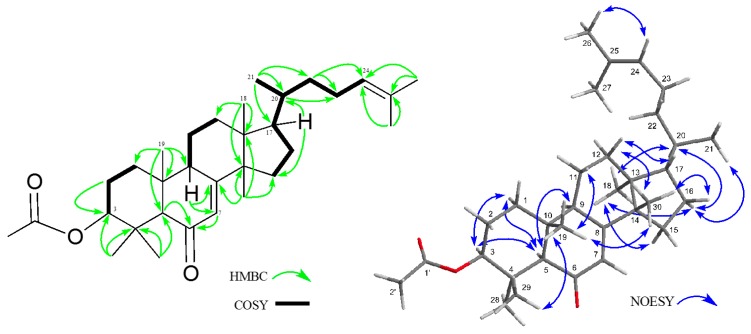
Key HMBC, COSY, and NOESY correlations for **3**.

Compound **3**, a colorless crystal, had the molecular formula of C_32_H_50_O_3_ (*m/z* 482.3764 [M]^+^, calcd 482.3760) by HREIMS. The IR spectrum and UV absorption band showed the presence of an ester group (ν_max_ 1731 cm^–1^) and an αβ-unsaturated six-membered ring ketone [ν_max_ 1664 cm^–1^, λ_max_ = 244.5 nm (log ε 3.99)]. The ^1^H- and ^13^C-NMR spectra (Table 1) exhibited signals assignable to five tertiary methyl groups (δ_H_ 0.82, 0.88, 1.05, 1.20 (6H) (each s)); a secondary methyl (δ_H_ 0.87 (d)), two vinyl methyls (δ_H_ 1.61, 1,69 (each s)), an acetyl (δ_H_ 2.07 (3H, s), δ_C_ 171.0 (s)); eight sp^3^ methylene, five sp^3^ methines including an oxymethine (δ_H_ 4.47 (dd)); four sp^3^ quaternary carbons, two trisubstituted olefins (δ_H_ 5.09 (tt), δ_C_ 124.8 (d), 131.2 (s)), (δ_H_ 5.69 (d), δ_C_ 124.8 (d), 170.8 (s)), and an αβ-unsaturated ketone (199.4 (s)). Cross peaks in the ^1^H-^1^H COSY spectrum are shown in bold lines in Figure 4. In the HMBC spectrum, these cross peaks were observed from Me-18 (δ_H_ 0.82 (s))/C-12, C-13, C-17; Me-19 (δ_H_ 0.88 (s))/C-1, C-9, C-10; Me-21 (δ_H_ 0.87 (d))/C-17, C-20, C-22; Me-26 and Me-27 (δ_H_ 1.69 and 1.61 (each 3H, s))/C-24 (δ_C_ 124.8 (d)), C-25 (δ_C_ 131.2 (s)); Me-28 and Me-29 (δ_H_ 1.20 (6H, s))/C-3 (δ_C_ 80.6 (d)), C-4, C-5; Me-30 (δ_H_ 1.05 (s))/C-8, C-13, C-14, C-15; H-7 (δ_H_ 5.69 (d))/C-5, C-6 (δ_C_ 199.4 (s)), C-8, C-9, C-14; H-24 (δ_H_ 5.09 (tt))/C-22, C-23, C-25 (δ_C_ 131.2 (s)), C-26, C-27. The relative configuration of **3** was elucidated on the basis of NOESY correlations (Figure 4). Intense NOESY correlations between Me-18 and H-16α; between Me-19 and Me-29, Me-30, and H-11β; between Me-30 and Me-19, H-11β, H-12β, H-16β and H-17; between H-3 and H-1α, and H-5α; between H-7 and H-15α, Me-19, and Me-30; between H-16α and Me-18, Me-21, and H-15α; between H-20 and H-16α and Me-18, indicated that the acetyl group at the C-3 position was in the β orientation, as H-17 was β-orientation. However, Me-18 and Me-21 were in the α orientation. More importantly, NOESY cross peaks for H-12β with H-17; Me-21 with H-16α and H-16β; and H-24 with H-12β, H-17 and Me-27, which suggested that **3** would be a euphane-type triterpene. Pettit et al. reported five euphane triterpenoids named Meliastatins **1**–**5** from *Melia dubia*, and the NOESY spectrum of Meliastatins resembles that of **3** [11]. The ^13^C-NMR chemical shift values of the side chain of **3** had similar literature data of butyrospermol [12] and kansenone [13]. Based on these results and the biogenetical consideration [14], **3** was established as 3β-acetoxyeupha-7,24-dien-6-one, isolated for the first time. Although euphane-type triterpenoids have been known for a long time, they are not present in abundant amounts in nature.

Macrophages may be a potential therapeutic target for inflammatory diseases [15]. Activated macrophages release pro-inflammatory mediators, such as NO, reactive oxygen species, interleukin-1 beta, tumor necrosis factor-alpha, and other inflammatory mediators, which play important roles in biological defenses. However, the overexpression of these mediators has been implicated in diseases such as osteoarthritis, rheumatoid arthritis, and diabetes, because the increased production of pro-inflammatory mediators has been shown to induce severe or chronic inflammation [15]. Ten triterpenoids and l-NMMA—an inducible nitric oxide synthase (iNOS) inhibitor—were evaluated for their inhibitory effects on NO production (Table 2). Among the compounds tested, **4**, **6**, **7**, **8**, and **10** exhibited NO inhibitory activities. Of these, **7** and **8** exhibited no cytotoxicity at 1–30 μM. Although **4**, **6,** and **10** exhibited some cytotoxicity at higher concentrations, they had similar inhibitory effects on NO production by triterpenoids from roots of *Taraxacum officinale* superior inhibitory activities to l-NMMA at non-toxic concentrations (**4** at 3–10 μM; **6** at 1 and 3 μM; **10** at 3 and 10 μM).

## 3. Experimental Section

### 3.1. General Procedures

Melting points were measured on a Yanagimoto micro-melting point apparatus (Yanagimoto, Kyoto, Japan) and were uncorrected. Optical rotations were measured using a JASCO DIP-1000 digital polarimeter (JASCO, Tokyo, Japan). IR spectra were recorded using a Perkin-Elmer 1720X FTIR spectrophotometer (Perkin-Elmer Inc., Wellesley, MA, USA). ^1^H- and ^13^C-NMR spectra were obtained on an Agilent VNMRS 600 spectrometer (Agilent Technologies, Santa Clara, CA, USA) with standard pulse sequences, operating at 600 and 150 MHz, respectively. CDCl_3_ was used as the solvent, and tetramethylsilane (TMS) as the internal standard. HREIMS were recorded on a JEOL-7000 mass spectrometer (JEOL, Tokyo, Japan). Column chromatography was performed over silica gel (70–230 mesh, Merck, Darmstadt, Germany), while medium pressure liquid chromatography (MPLC) was conducted with silica gel (230–400 mesh, Merck). HPLC was run on a JASCO PU-1586 instrument (JASCO, Tokyo, Japan) equipped with a differential refractometer (RI 1531). Fractions obtained from column chromatography were monitored by thin layer chromatography (TLC) (silica gel 60 F_254_, Merck).

### 3.2. Plant Material

The roots of *T. officinale* (Compositae) were collected in Takatsuki city, Osaka, Japan in April 2014. A voucher specimen (TR-01) was deposited in the Herbarium of the Laboratory of Medicinal Chemistry, Osaka University of Pharmaceutical Sciences.

### 3.3. Isolation of Compounds ***1***–***3***

The roots of *T. officinale* (5.81 kg) were extracted with MeOH at 60 °C for 10 days, and the dried extract obtained (890 g) was partitioned between EtOAc and H_2_O. The organic layer (290 g) was condensed in vacuo and subjected to column chromatography (silica gel (3 kg); hexane:EtOAc (5:1→1:1→0:1) and EtOAc:MeOH (1:1→0:1)), affording residues A (Fr. No. 1 and 2, 0.72 g), B (Fr. No. 3, 2.42 g), C (Fr. No. 4 and 5, 5.17 g), D (Fr. No. 6–10, 3.37 g), E (Fr. No. 11, 0.44 g), F (Fr. No. 12, 1.84 g), G (Fr. No. 13, 4.87 g), H (Fr. No. 14, 6.29 g), I (Fr. No. 15, 4.64 g), J (Fr. No. 16, 1.89 g), K (Fr. No. 17–20, 1.46 g), L (Fr. No. 21–27, 7.02 g), M (Fr. No. 28–40, 1.98 g), N (Fr. No. 41–52, 2.32 g), O (Fr. No. 53–66, 42.48 g), P (Fr. No. 67–78, 1.49 g), Q (Fr. No. 79–84, 0.54 g), R (Fr. No. 85–94, 30.57 g), and S (Fr. No. 95–108, 5.68 g). Residue C was rechromatographed over silica gel to give a triterpene fraction, which was subjected to HPLC (ODS, 95% MeOH) to afford compounds **1** (1.38 mg) and **9** (1.12 mg) [16]. Residue D was rechromatographed over silica gel to give a triterpene fraction, which was subjected to HPLC (ODS, 95 % MeOH) to afford compounds **2** (1.60 mg), **3** (2.40 mg), **5** (1.61 mg) [17], **8** (1.39 mg) [18], and **10** (2.82 mg) [19]. Residue L was rechromatographed over silica gel to give a triterpene fraction, which was subjected to HPLC (ODS, 95% MeOH) to afford compounds **4** (20.23 mg) [5] and **7** (1.06 mg) [5]. Residue O was rechromatographed over silica gel to give a triterpene fraction, which was subjected to HPLC (ODS, 95% MeOH) to afford compound **6** (1.94 mg) [20].

### 3.4. Analytical Data

*18β,19β-Epoxy-21β-methoxylupan-3β-yl acetate* (**1**): Colorless amorphous; ${\left[\alpha \right]}_{D}^{25}$ +36.2° (*c* 0.04, EtOH); HREIMS *m*/*z*: 514.4022 [M]^+^ (C_33_H_54_O_4_, calcd. for 514.4022); IR (KBr) ν_max_ cm^−1^: 2956, 2925, 1739 (O-C=O), 1456, 1381, 1367, 1246; ^1^H- and ^13^C-NMR, see Table 1. EIMS *m*/*z* (relative intensity (rel. int.)): 514 [M]^+^ (100), 483 (26), 416 (45), 325 (4), 233 (6), 191 (9), 135 (12).

*3β-Acetoxybauer-7-en-6-one* (**2**): Colorless crystals; mp 110–112 °C; ${\left[\alpha \right]}_{D}^{25}$ +21.9° (*c* 0.1, EtOH); HREIMS *m*/*z*: 482.3759 [M]^+^ (C_32_H_50_O_3_, calcd for 482.3760); UV λ_max_ (EtOH) nm (log ε): 245.0 (3.97); IR (KBr) ν_max_ cm^−1^: 2954, 2869, 1733, and 1247 (O-C=O), 1665 (O=C-C=C), 1466, 1388; ^1^H- and ^13^C-NMR, see Table 1. EIMS *m*/*z* (rel. int.): 482 [M]^+^ (100), 422 [M − HOAc]^+^ (48), 407 (31), 379 (30), 300 (45), 219 (46), 206 (44), 203 (42), 191 (35), 161 (40).

*3β-Acetoxyeupha-7,24-dien-6-one* (**3**): Colorless crystals; mp 140–143 °C; ${\left[\alpha \right]}_{D}^{25}$ −38.1° (*c* 0.04, EtOH); HREIMS *m*/*z*: 482.3764 [M]^+^ (C_32_H_50_O_3_, calcd. for 482.3760); UV λ_max_ (EtOH) nm (log ε): 244.5 (3.99); IR (KBr) ν_max_ cm^−1^: 2958, 1731, and 1247 (O-C=O), 1664 (O=C-C=C), 1437, 1384; ^1^H- and ^13^C-NMR, see Table 1. EIMS *m*/*z* (rel. int.): 482 [M]^+^ (59), 467 (56), 422 [M − HOAc]^+^ (26), 407 (34), 369 (100), 300 (43), 217 (16), 206 (44), 189 (17), 161 (45).

### 3.5. Determination of RAW264.7 Cell Proliferation

RAW264.7 cell proliferation was examined in accordance with a previously reported method [21]. Briefly, RAW264.7 cells (5 × 10^4^ cells in 100 μL) were seeded on a 96-well microplate and incubated for 24 h. Dulbecco's modified eagle's medium (DMEM)-containing test samples (100 μL total volume, final concentration of 30, 10, 3, or 1 μM) dissolved in dimethyl sulfoxide (DMSO, final concentration 0.2%) was added. After being treated for 24 h, thiazolyl blue tetrazolium bromide (MTT) solution was added. After a 3-h incubation, 20% sodium dodecyl sulfate in 0.1 M HCl was added to dissolve the formazan produced in the cells. Absorbance in each well was read at 570 nm using a microplate reader. The optical density of vehicle control cells was assumed to be 100%.

### 3.6. Inhibitory Assay of NO Production

An inhibitory assay of NO production was examined in accordance with a previously reported method [22] with minor modifications. Briefly, RAW264.7 cells (5 × 10^4^ cells in 100 μL) were seeded on a 96-well microplate and incubated for 24 h. DMEM-containing test samples (100 μL total volume, final concentration of 30, 10, 3, or 1 μM) dissolved in DMSO (final concentration 0.2%) and LPS (final concentration of 5 μg/mL) was added. After being treated for 24 h, the supernatant of the culture medium was transferred to another 96-well microplate, and 50 μL of 0.15% *N*-(1-naphtyl)ethylenediamine in H_2_O and 1.5% sulfanilamide in 7.5% phosphoric acid were then added. After being incubated for 30 min, absorbance in each well was read at 570 nm using a microplate reader. The optical density of vehicle control cells was assumed to be 100%

## 4. Conclusions

A novel lupane-type triterpenoid (**1**), as well as a novel bauerane-type triterpenoid (**2**) and a novel euphane-type triterpenoid (**3**) were isolated from the roots of *T. officinale* (Compositae), together with seven known triterpenoids (**4**–**10**). Their structures were elucidated by spectroscopic analyses. In the NO inhibitory assay, Compounds **4**, **6**, and **10** exhibited similar NO inhibitory activities (IC_50_ **4**: 16.9 μM; **6**: 17.0 μM; **10**: 18.4 μM) to l-NMMA (IC_50_ 23.9 μM). These compounds did not exhibit cytotoxicity at an effective concentration (**4** and **10** at 1–10 μM; **6** at 1 and 3 μM). These results suggest that compounds **4**, **6**, and **10** have potential as anti-inflammatory disease agents.

## Figures and Tables

**Table 1:** ^1^H- (600 MHz) and ^13^C- (150 MHz) NMR data of compounds **1**–**3**.

Position		1				2				3		
	^1^H (*J*, Hz)		^13^C		^1^H (*J*, Hz)		^13^C		^1^H (*J*, Hz)		^13^C	
1α	1.02	m	38.3	t	1.46	m	36.3	t	1.49	m	36.6	t
1β	1.68	m			1.68	m			1.70	m		
2	1.64	m (2H)	23.7	t	1.65	m (2H)	23.3	t	1.65	m (2H)	23.3	t
3	4.47	dd (5.3, 11.4)	80.9	d	4.47	dd (4.1, 11.7)	80.6	d	4.47	dd (4.1, 11.7)	80.6	d
4			37.8	s			36.9	s			37.0	s
5	0.81	m	55.3	d	2.20	s	65.1	d	2.20	s	65.2	d
6α	1.54	m	18.2	t			199.8	s			199.4	s
6β	1.41	m										
7	1.41	m (2H)	33.79	t	5.84	d (3.0)	123.9	d	5.69	d (3.0)	124.8	d
8			41.0	s			170.3	s			170.8	s
9	1.38	dd (4.2, 12.0)	49.8	d	2.73	ddd (3.0, 6.8, 12.6)	49.7	d	2.72	ddd (3.0, 6.2, 13.2)	50.3	d
10			37.0	s			43.5	s			43.6	s
11	1.51	m	20.55	t	1.74	m	16.3	t	1.72	m	17.6	t
					1.55	m			1.56	m		
12	1.44	m	22.9	t	1.70	m	31.6	t	1.78	m	32.7	t
					1.55	m			1.87	m		
13	2.48	dd (4.1, 11.8)	33.75	d			37.3	s			43.0	s
14			44.4	s			42.8	s			52.4	s
15α	1.10	m	26.6	t	1.46	m	28.07	t	1.56	m	32.8	t
15β	1.79	td (4.1, 13.5)			1.57	m			1.50	m		
16α	1.59	m	31.5	t	1.54	m	37.2	t	1.35	m	27.9	t
16β	1.40	m			1.27	m			1.99	m		
17			41.2	s			32.0	s	1.56	m	52.7	d
18			77.0	s	1.33	brs	54.7	d	0.82	s	22.0	q
19			71.9	s	1.17	m	35.4	d	0.88	s	14.3	q
20	1.91	m	29.4	d	1.58	m	31.9	d	1.43	m	35.5	d
21	3.77	d (5.9)	83.2	d	A 1.20	m	29.1	t	0.87	d (6.5)	18.4	q
					B 1.59	m						
22	α 1.18	dd (6.4, 13.5)	39.3	t	A 1.19	m	31.2	t	A 1.01	m	35.1	t
22	β 1.32	m			B 1.59	m			B 1.57	m		
23	0.85	s	27.9	q	1.20	s	28.11	q	A 1.88	m	25.3	t
									B 2.05	m		
24	0.84	s	16.5	q	1.19	s	15.9	q	5.09	tt (1.5, 14.1)	124.8	d
25	0.88	s	16.2	q	0.88	s	14.2	q			131.2	s
26	1.075	s	16.0	q	1.06	s	21.3	q	1.69	s	25.7	q
27	1.05	s	14.7	q	0.96	s	22.7	q	1.61	s	17.7	q
28	1.083	s	23.4	q	1.06	s	37.8	q	1.20	s	16.0	q
29	1.11	d (7.4)	20.63	q	1.07	d (8.0)	25.6	q	1.20	s	28.2	q
30	1.11	d (7.4)	18.4	q	0.92	d (5.9)	22.5	q	1.05	s	24.9	q
21-OMe	3.22	s	56.5	q								
1′			171.0	s			171.0	s			171.0	s
2′	2.04	s	21.3	q	2.07	s	21.2	q	2.07	s	21.2	q

**Table 2:** Inhibitory effects of NO production by triterpenoids from roots of *Taraxacum officinale*.

	Produced NO % (Cell Viavility %) ^a^				
	1 μM	3 μM	10 μM	30 μM	IC_50_ (μM)
**1**	94.8 ± 1.2	101.4 ± 0.7	90.5 ± 2.4	95.7 ± 4.6	>30
	(95.0 ± 0.7)	(104.2 ± 1.3)	(102.6 ± 0.7)	(107.4 ± 2.0)	
**2**	95.6 ± 2.7	100.7 ± 2.6	86.5 ± 1.4 **	71.4 ± 1.8 **	>30
	(100.0 ± 1.3)	(102.2 ± 0.3)	(105.1 ± 0.3)	(105.7 ± 2.0)	
**3**	100.6 ± 2.6	93.7 ± 1.4	93.3 ± 1.6	93.1 ± 0.9	>30
	(100.0 ± 1.4)	(94.6 ± 8.2)	(104.2 ± 0.7)	(103.8 ± 1.0)	
**4**	83.6 ± 0.9 **	76.8 ± 2.5 **	66.0 ± 0.8 **	35.4 ± 2.2 **	16.9
	(98.4 ± 0.9)	(97.9 ± 0.3)	(92.7 ± 1.0)	(49.5 ± 0.7)	
**5**	110.3 ± 5.2	106.1 ± 3.2	94.9 ± 4.3	92.7 ± 4.7	>30
	(98.4 ± 3.1)	(94.8 ± 3.9)	(90.7 ± 1.8)	(88.9 ± 2.3)	
**6**	83.4 ± 1.5 **	75.0 ± 3.2 **	65.4 ± 0.5 **	36.3 ± 0.6 **	17.0
	(99.8 ± 0.1)	(93.7 ± 1.1)	(76.7 ± 0.7)	(66.7 ± 1.2)	
**7**	101.0 ± 1.0	101.0 ± 2.5	89.8 ± 1.1 **	66.8 ± 0.8 **	>30
	(98.3 ± 2.6)	(103.0 ± 2.4)	(99.7 ± 3.8)	(99.8 ± 3.0)	
**8**	102.9 ± 0.7	105.0 ± 3.1	90.2 ± 7.1	64.1 ± 3.3 **	>30
	(99.9 ± 0.1)	(100.4 ± 0.1)	(100.5 ± 0.2)	(103.0 ± 1.6)	
**9**	98.4 ± 3.9	100.4 ± 1.6	109.1 ± 2.0 **	108.2 ± 4.7 *	>30
	(99.3 ± 0.2)	(98.8 ± 0.2)	(99.1 ± 0.4)	(99.9 ± 0.6)	
**10**	93.8 ± 1.8	81.6 ± 3.7 **	67.5 ± 2.4 **	37.0 ± 1.8 **	18.4
	(108.3 ± 4.7)	(100.4 ± 0.2)	(100.2 ± 0.1)	(2.6 ± 0.4)	
l-NMMA ^b^	93.3 ± 2.2	91.4 ± 0.8	68.9 ± 4.5 **	43.1 ± 1.1 **	23.9
	(101.5 ± 0.9)	(101.9 ± 0.4)	(98.5 ± 0.9)	(109.4 ± 0.5)	

^a^ Produced NO (%) and cell viability (%) were determined based on the absorbance at 570 nm, respectively, by comparison with values for DMSO (100%). Each value represents the mean ± standard error (S.E.) of four determinations. Significant differences from the vehicle control group shown as * *p* < 0.05 and ** *p* < 0.01; ^b^ Positive control.
